# A systematic review and meta-analysis of psychological treatments to improve sleep quality in university students

**DOI:** 10.1371/journal.pone.0317125

**Published:** 2025-02-13

**Authors:** Michelle Tadros, Jill M. Newby, Sophie Li, Aliza Werner-Seidler

**Affiliations:** 1 The Black Dog Institute, Prince of Wales Hospital, Randwick, NSW, Australia; 2 School of Psychology, UNSW Sydney, Sydney, NSW, Australia; 3 Faculty of Medicine, UNSW Sydney, Sydney, NSW, Australia; Chiba Daigaku, JAPAN

## Abstract

**Objective:**

This paper reviews the literature evaluating psychological treatments to improve sleep quality in young adult university students.

**Method:**

Participants (N = 6179) were young adult (aged 18–30 years) university students. Databases (PubMed, PsychInfo, EMBASE and Medline) were searched for randomized controlled trials evaluating psychological treatments for sleep disturbance in university students. The search date was 20 September 2024.

**Results:**

22 original trials met inclusion criteria. Meta-analysis showed that psychological interventions outperformed control groups (n = 14) on improving sleep quality (*g* = 0.50, 95%CI:0.26–0.73). There were significantly different effect sizes found between studies that evaluated cognitive behaviour therapy for insomnia (CBT-I; n = 6, *g* = 0.72, CI: 0.43–1.02) versus studies that evaluated mindfulness interventions (n = 5, *g* = 0.16, 95% CI: -0.18–0.51).

**Conclusions:**

Psychological treatments improve sleep quality for young adult university students. While CBT-I showed larger effect sizes than interventions focused on mindfulness, further research is needed to verify if this reflects a true difference in the efficacy of the interventions.

## Introduction

Advances in sleep science have shown that persistent poor sleep, or insomnia, predisposes individuals to develop mental illnesses such as depression and anxiety [1–4]. Sleep difficulties can also worsen comorbid mental illness: increasing the likelihood of depression relapse [5]; prolonging the course of depression [6]; and blunting treatment effects [7]. As a consequence, mental health clinicians and physicians now consider the effective treatment of sleep disturbance to be a priority [8]. This greater awareness of the fundamental role sleep plays in maintaining physical and mental health, and the detrimental impact of sleep difficulties on mental health, underscores the need for effective psychological interventions that improve sleep quality.

The literature on treating sleep difficulties in general adult populations has shown that psychological interventions are beneficial [9–11]. They are at least as effective as pharmacological treatments for reducing sleep onset latency, improving sleep efficiency and self-reported sleep quality [12], and therefore recommended as first line treatments [13]. There are a diverse range of psychological treatments available for this age group, including mindfulness meditation, relaxation, sleep hygiene and Cognitive Behaviour Therapy for Insomnia (CBT-I). The research evaluating psychological treatments, however, has rarely involved young adults or university students with participants typically being middle aged and older adults [14]. Consequently, it is unclear if the existing treatments are helpful for tertiary education students, who are typically young adults in circumstances unique from other adults. For instance, students often have living arrangements that make regular sleep routines difficult to sustain. Many students reside in dorms, residential halls or share houses where late night social interactions may encourage some students to delay their bedtime [15]. Alternatively, the noise made by other students engaged in social activities can be a disruption to students who wish to sleep. Students living with their family of origin, face different challenges to sleep. In these cases, students may spend large amounts of time in their bedroom for activities that do not include sleep such as studying and socialising online, making it difficult for students to develop a conditioned association between being in their bed with feeling sleepy [15, 16].

Given the circumstances of students is often not conducive to healthy sleep patterns, it is not surprising that the prevalence rate of insomnia in university students is higher than what is reported in the general population, where the point prevalence is approximately 6% [17]. For example, a systematic review of studies exploring insomnia in university students, found 18.5% met criteria for insomnia disorder using validated scales [18]. In addition, there are many young people who experience suboptimal sleep duration or quality. but do not meet criteria for insomnia disorder. Studies with young adult university students report between 43% and 66% are poor sleepers [16, 19].

Not only do university students experience elevated rates of sleep problems, but they also have high rates of mental illness with 31% reporting a mental health disorder in the past twelve months [20]. There is also evidence that the prevalence of mental health disorders in this population has been steadily increasing; Lipson et al. [21] found the proportion of students with a mental health disorder rose from 21.9% in 2007 to 35.5% in 2017. Improving sleep quality in this population is important for many reasons. For example, sleep disturbance is an independent risk factor for subsequent mental illness [4] and suicidal behaviour [1–4]. Furthermore, untreated sleep disturbance may serve to maintain comorbid depression [22] and increase risk of relapse [23].

There have been two systematic reviews that examined the efficacy of different forms of psychological treatments to improve sleep in young adults, and included only randomised controlled trials (RCTs) [24, 25]. Both reviews found psychological interventions improved sleep with a moderate effect size (g = 0.53; g = 0.61, respectively), however, a significant limitation is that these reviews included only studies with non-active control groups (e.g., no intervention, waitlist). The use of non-active control groups can lead to an overestimation of intervention effect sizes [26], as such study designs do not control for participant expectations or the experience of receiving treatment including contact with health care providers. These factors not related specifically to the intervention being tested can contribute to symptom improvement. Studies that make use of active controls (such as psychoeducation about sleep, sleep hygiene information) are a valuable source of information that offer a more conservative and potentially more accurate estimate of intervention effects. Since the most recent review [24] conducted their search in April 2020, several important studies have been published [27–32], four of these [28, 30–32] have investigated the effect of mindfulness on sleep quality in students. Previously, there were very few RCTs examining mindfulness for sleep [24, 25]. The addition of these new studies potentially impacts treatment recommendations regarding the use of mindfulness and warrants the need for an updated review.

To extend on previous reviews, we sought to conduct an updated systematic review and meta-analysis of the relevant published RCTs of psychological interventions to improve sleep quality in university students, including studies with both active and non-active control groups. Studies reporting measures of comorbid psychopathology (e.g. anxiety and depression) and daytime functioning (such as quality of life and sleepiness) were noted in the narrative summary. These measures give an indication of the ways in which psychological treatments to improve sleep can impact daily life, giving a more complete picture of the utility of such interventions [33].

The primary aim of this review was to synthesise the RCT literature examining psychological treatments to improve sleep quality in young adult university students and use meta-analytic techniques to examine their efficacy relative to control groups in improving sleep quality. A secondary aim was to compare the effect sizes of different psychological approaches to treating sleep disturbance in university students. A diverse range of psychological interventions exist; however, it is unknown how specific treatment approaches compare to others in this population. In keeping with the literature on treating sleep difficulties in adults, it was hypothesized that psychological interventions will be effective at improving sleep in young adult university students, relative to a control group.

## Method

### Search strategy

The review was prospectively registered with PROSPERO (registration number: CRD42020177375) where the protocol can be viewed. A comprehensive search of the databases PubMed, PsychInfo, EMBASE and Medline was conducted on 20 September 2024. No restrictions were placed on the date of publication when searching databases, all available studies were included. The search terms used were related to sleep difficulty (e.g. insomnia, sleep disturbance, sleep disorder), psychological treatment (e.g. cognitive behaviour therapy, behaviour therapy, mindfulness) and young adults (e.g. youth, university students, young people). See supplementary material for further details on the search terms used. The reference lists from all related articles were also hand searched for relevant articles on sleep treatments for young adults. Initially, titles and abstracts were screened by one author (MT). Selected abstracts and full text articles were screened independently by MT, SL and AWS and a final set of articles were selected for inclusion in the review. Each independent reviewer cross checked their selections with other reviewers and the final list was checked by JN. Any disagreements between the independent reviewers (MT, SL and AWS) were resolved with JN’s contribution.

### Inclusion/exclusion criteria: PICOS

#### Participants

Studies were eligible for inclusion if they included participants aged on average between 18–30 years. This age range was selected as the population of interest in this study is young adult university students, and there is very little research reporting on sleep outcomes in this age group. It was not essential that studies had sleep disturbance symptom criteria for participants to enter the study. Studies were excluded if the majority of participants had severe mental or physical health comorbidities (e.g. PTSD, chronic pain, cancer, chronic life-threatening illness, inpatient psychiatric patients). Studies were also excluded if participants were drawn from forensic or veteran populations because these groups would not be comparable to a typical university student population of young adults.

#### Interventions

To be included, studies were required to test a psychological intervention, such as CBT-I, mindfulness, relaxation or sleep hygiene that was focused on treating sleep difficulties. Studies that evaluated interventions such as music therapy and physical therapies (e.g. Tai Chi and Yoga) were not considered to be primarily psychological interventions and were not eligible for inclusion.

#### Controls

Only studies with control groups were included. These could be active (e.g. educational groups, sleep hygiene or sleep education, general health advice) or non-active control groups such as waitlist or care as usual groups.

#### Outcomes

Studies were included that reported outcome data on at least one sleep variable, such as self-reported insomnia severity, sleep onset latency, or subjective sleep quality. Other relevant outcomes were measures of psychopathology and daytime functioning, such as wellbeing, quality of life and daytime sleepiness.

#### Studies

Studies were included if they were written in English, published in a peer reviewed journal and were randomized controlled trials. It has been demonstrated repeatedly that the inclusion of studies with small sample sizes can distort the findings of meta-analytic reviews by artificially inflating overall effect sizes [34]. As such, studies with group sizes lower than 20 per group were also excluded from the review.

### Data extraction

The following study characteristics were extracted by MT and independently checked by second author (AWS or SL): 1) author, year of publication, and country of study, 2) participant age, mean, SD and range, 3) insomnia symptom criteria for inclusion, 4) intervention type, treatment delivery mode (face to face, online, group), number of sessions and session duration, 5) time points of data collection, 6) number of participants (total and per group), 7) control group type, 8) sleep measures and 9) other outcome measures (such as measures of quality of life, sleepiness, dysfunctional beliefs about sleep, sleep hygiene knowledge).

### Quality assessment

The quality of studies was determined using guidelines from Cochrane’s Risk of Bias Tool 2 [35]. When evaluating the quality of each study the following five sources of bias were considered. 1) Risk of bias arising from the randomization process—this entails considering whether participants were randomly allocated to groups and if allocation was concealed. 2) Risk of bias due to deviations from the intended interventions—this refers to the awareness participants or carers have of the group they are assigned to. 3) Risk of bias due to missing outcome data—methods of analysis to correct for missing data should be used where more than 5% of data are missing. 4) Risk of bias in measurement of the outcome–the use of a poorly validated measure. 5) Risk of bias in selection of the reported result–the analysis should be consistent with the researchers plans prior to the availability of data. MT and either JN or AWS completed the risk of bias assessments independently and any discrepancies were resolved through discussion by the three raters.

### Meta-analyses

An effect size (Hedges g) was derived from comparisons of the pre and post mean scores and standard deviations in the intervention and control groups in each study. Psychological interventions varied across studies; with the predominant approaches being sleep hygiene, CBT-I and mindfulness. Some studies evaluating CBT-I and mindfulness used sleep hygiene as a control group for comparison, these were excluded from the meta-analysis so that sleep hygiene was not appearing as both a treatment and a control in the meta-analysis. One study [36] had two control groups, a waitlist control and placebo control, only the waitlist control was included in the meta-analysis as this data was of primary interest for the purposes of the meta-analysis. Effect sizes of 0.2, 0.5 and 0.8 refer to small, moderate and large effect sizes respectively [37]. To calculate a pooled mean effect size, we used the program Comprehensive Meta-Analysis (version 4.0, Biostat Inc.). Given there is variability across the interventions, a considerable level of heterogeneity (measured by *I*^*2*^) is expected, so we calculated the mean effect sizes, and 95% confidence intervals using a random effects model. The random effects model assumes that the true effect size varies from one study to the next, and that the studies in our analysis represent a random sample of effect sizes that could have been observed. The summary effect is our estimate of the mean of these effects [38]. These results will be presented in the form of forest plots. The I^2^ statistic was used as a measure of heterogeneity.

### Subgroup analysis

Planned subgroup analyses examined intervention type, comparing the effect size of studies that evaluated CBT-I with studies that evaluated the effect of mindfulness. A random effects model was used to estimate effect sizes within groups and a fixed effects model was used to compare effect sizes between subgroups.

### Testing for publication bias and dealing with publication bias

To test for publication bias, we inspected the funnel plot on sleep quality, the only outcome measure that was available in all the studies selected for the meta-analysis [39]. In addition, we also conducted Duval and Tweedie’s Trim and Fill procedure [40, 41] within Comprehensive Meta-Analysis, which yields an adjusted effect size that takes into account the publication bias observed within the funnel plot. This procedure corrects for the variance of the effects and provides a best estimate of the unbiased effect size.

## Results

Data extracted from included studies and used in meta-analysis is available from the corresponding author.

### Selection of studies

**Fig 1:**
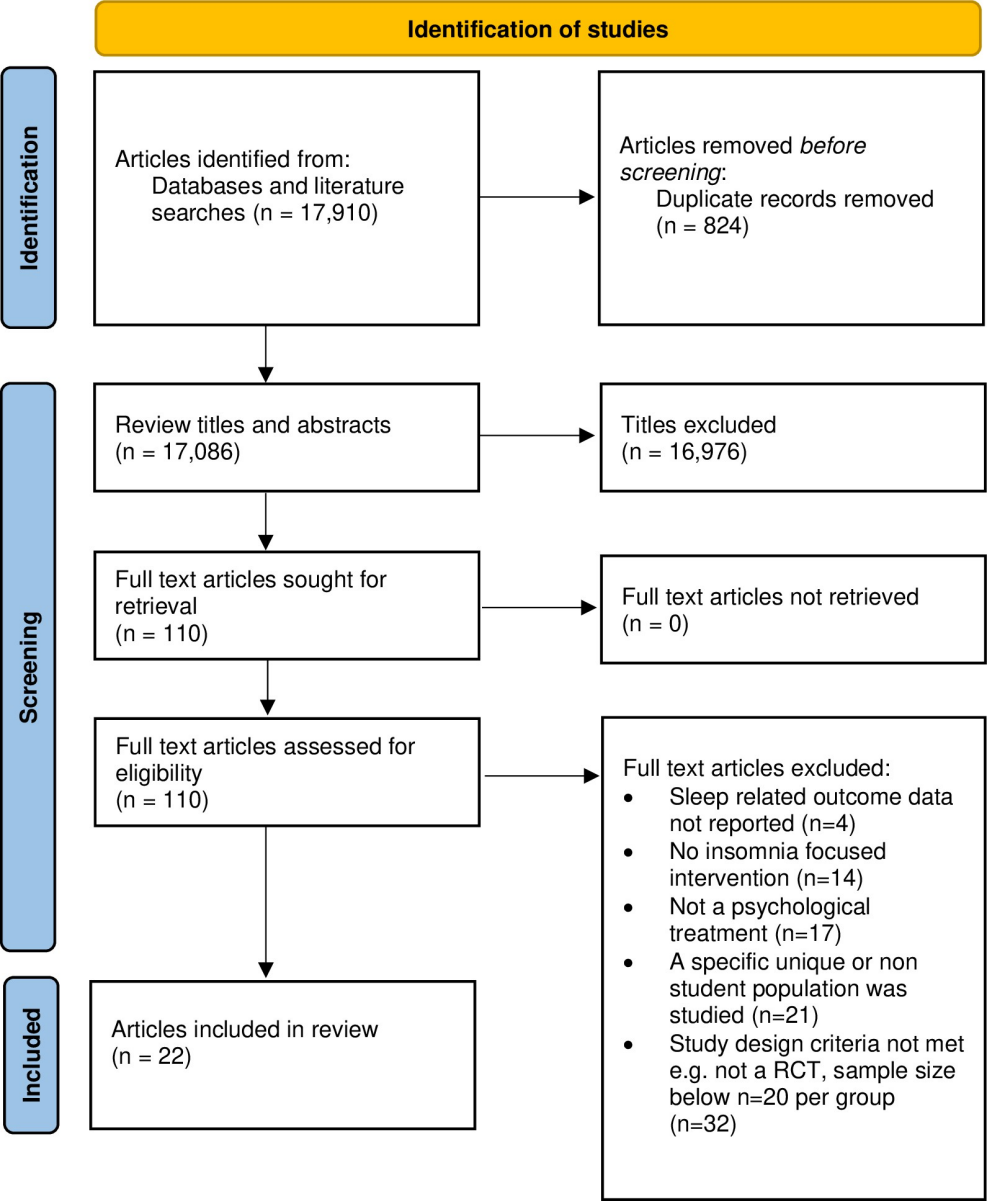
PRISMA flow chart.

Fig 1 presents the flowchart of the search and selection of included studies. Database and reference list searches yielded 17,910 titles. After the removal of 824 duplicates, 17,086 titles and abstracts were screened. After reviewing 110 full text articles, 22 original trials met the inclusion criteria.

### Characteristics of included studies

#### Participants

A total of 6179 participants (intervention groups n = 3125, control groups n = 3054) were included in the review. Studies were published from the year 2000 to 2024. All studies included university student samples.

#### Study location

Most studies were conducted in the USA (k = 11), four studies from China, three from the UK and there was one study each from Australia, Brazil, Germany and Japan.

#### Criteria for entry

The majority of studies (64%) had no sleep disturbance symptom criteria for study entry, while the remaining studies (36%) had different sleep symptom criteria for study entry ranging from a requirement to report some concerns with sleep to meeting full diagnostic criteria for insomnia. A full account of study characteristics can be found in Table 1.

**Table 1:** Study characteristics.

First Author, Year Country	Participants Mean Age (SD, range)	Insomnia Sx Criteria for Entry	Intervention Type, Modality, Number Sessions X duration of session (minutes)	Time Points Data Collection	N	Control Group	Insomnia Measures	Other Measures	
Barber 2017 USA	20 (4.84, range not reported)	None	Sleep Hygiene, Power point slide presentation, 1 x 23min	Baseline, post treatment	N = 78 Treatment = 43 Control = 35	Care as usual	SQS; SHI, Objective by actigraphy: TST, NWAK, SE. Self report: TST	Technology Use Questionnaire	
Classen 2022 Germany	Treatment: 25.97 (3.94, range not reported) Control: 27.84 (6.56, range not reported)	PSQI >5	CBT-I with hypnosis, group face to face, 6 x 100min	Baseline, post treatment	N = 54 Treatment = 35 Control = 19	Waitlist	PSQI	D2 Test of attention, TAP, VLMT, WMS	89% (completion means attendance at 67% of sessions)
Denis 2020 UK	Treatment: 19.73 (2.94, range not reported) Control: 20.22 (5.69, range not reported)	None	CBT-I, online, 6 x 20min	Baseline, mid treatment, post treatment, follow-up	N = 199 Treatment = 99 Control = 100	Puzzles	SCI, PSQI	STAI-S, PSS, PSAS, DBAS, The list of life threatening experiences, PMH, Mood and feelings questionnaire, Specific Psychotic experiences Questionnaire, Sleep disturbances related to trauma, chronotype.	
Freeman 2017 UK	24.7 (7.7, range not reported)	SCI <17	CBT-I, Online, 6 x 20min	Baseline, mid treatment, post treatment, follow-up	N = 3755 Treatment = 1891 Control = 1864	Care as usual	SCI-8, ISI, Disturbing dreams and nightmares index	WEMWBS; WSAS; PHQ-9; Green et al Paranoid Thought Scale; SPEQ; Prodromal Questionnaire; GAD-7; Altman Mania Scale; Mental Health Diagnosis (%), taking psychiatric medication (%), using mental health services (%)	
Fucito 2017 USA	20.52 (1.31, range not reported)	Report concerns about sleep, and at least 1 heavy drinking occasion in the past month	CBT-I with alcohol education principles, 4 x?	Baseline, post treatment, 3 month follow-up	N = 42 Treatment = 21 Control = 21	Sleep hygiene	PSQI, Objective by actigraphy: TST, SE	PROMIS-SRI SF, AUDIT, BYAACQ, DDQ	
Gallo 2023 Brazil	25 (6.2, range 18–41)	None	Mindfulness, group face to face, 8 x 90min	Baseline, weekly measures, post treatment.	N = 136 Treatment = 71 Control = 65	Waitlist	ISI	PHQ-9, STAI-S, PSS	
Gao 2014 China	20.49 (2.46 range not reported)	PSQI > 7	Relaxation and stimulus control, face to face lecture, 3 x 45min	Baseline, post treatment	N = 84 Treatment = 42 Control = 42	Care as usual	PSQI	None	
Gellis 2013 USA	Not reported but participants were introductory psychology students at university	1) ISI> 8; 2)insomnia sx > 1 month; 3) WASO, SOL, EMA > 30 mins 3 nights a week for at least 1 month	Cognitive therapy, face to face lecture, 1 x 30min	Baseline, post treatment	N = 60 Treatment = 31 Control = 29	Sleep hygiene	ISI	None	
Gipson 2019 USA	20 (range 18–26)	None	Sleep hygiene, Text message, 12 text messages, 2 per week	Baseline, post treatment	N = 96 Treatment = 52 Control = 44	General health advice	PSQI	None	
Greeson 2014 USA	25.4 (5.7, 18–31)	None	Mindfulness, face to face lecture, 4 x 75min	Baseline, post treatment	N = 90 Treatment = 45 Control = 45	Waitlist	MOS SLP9	None	
Hall 2018 China	22.3 (2.63, range not reported)	None	Mindfulness, face to face lecture, 2 x?	Baseline, mid treatment, post treatment	N = 101 Treatment = 76 Control = 25	Waitlist	PSQI	DASS 21	
Hershner 2018 USA	21.9 (4.1, range not reported)	None	Sleep hygiene, online, 1 x 20min	Baseline, post treatment	N = 549 Treatment = 254 Control = 295	Care as usual	PSQI	ESS, PHQ, GHQ	
Jones 2019 USA	range: 18–19 years of age	None	Sleep hygiene, Text message, 49 texts in 6 weeks	Baseline, post treatment	N = 156 Treatment = 53 Control = 103	Waitlist	PSQI	None	
Kloss 2015 USA	21.11 (2.43, range not reported)	None	CBT-I, face to face lecture, 2 x 90min	Baseline, post treatment	N = 120 Treatment = 63 Control = 57	Sleep hygiene	PSQI, ISI, self report SOL	None	
Mairs 2015 Australia	20.7 (5.8, 17–49)	None	Planning with sleep hygiene, email, 1 x 30min	Baseline, post treatment	N = 92 Treatment = 49 Control = 43	Sleep hygiene	PSQI, ISI	None	
Means 2000 USA	21.2 (5.6, 17–44)	ICSD Insomnia criteria	Relaxation, face to face lecture, 3 x 20min	Baseline, post treatment	N = 57 Treatment = 28 Control = 29	Unmedicated waitlist	By self report: SOL, WASO, SE	DBAS, ESS, FSS, IIS, PSWQ,	
Mirabito 2022 USA	21.39	None	Mindfulness, telehealth groups, 4 x sessions (time length not reported)	Baseline, post treatment	N = 111 Treatment = 58 Control = 53	Waitlist	PSQI	FIPAQ, PWS, PSWQ, PANAS, PSS, DASS 21	
Morris 2016 UK	20.55 (1.9, range not reported)	None	CBT-I, Anxiety online, 6 x 20min	Baseline, post treatment	N = 138 Insomnia Treatment = 48 Anxiety Treatment = 43 Control = 47	Waitlist	PSQI	BDI, STAI-S	
Okajima 2022 Japan	19.56 (1.86)	ISI > 10	CBT-I, email, 8 x 30 minutes	Baseline, post treatment	N = 41 Treatment = 21 Control = 20	Sleep monitoring	ISI, SHPS, DBAS, FIRST, PSAS	DASS 21	
Pickett 2022 USA	21.64	Excluded Participants with Insomnia or psychological disorders	Mindfulness, Relaxation, online audio file, 5 x 15 minutes for 4 weeks	Baseline, post treatment	N = 114 Mindfulness Treatment = 37 Relaxation Treatment = 37 Control = 40	Care as usual	SCI, PSAS	SRDI, FFMQ-SF, PSS	
Wu 2023 China	Treatment—19.7 (2.28) Control– 19.8 (2.41)	None, only participants with suicidal ideation were included.	Mindfulness, face to face groups, 1 x 30 minute lecture, 1 x 15 minute session per week for 4 weeks	Baseline, post treatment	N = 60 Treatment = 30 Control = 30	Waitlist	PSQI	SSI, PSS	
Zhu 2024 China	18.87 (0.86, range not reported)	PSQI > 6	Group face-to-face CBT-I, digital CBT-I, 6 x 90min	Baseline, post treatment	N = 93 CBT-I n = 28 Digital CBT-I n = 30 Education = 31	Education	PSQI	DBAS, PSAS, APS	

APS–Arousal Predisposition scale, AUDIT–Alcohol Use Disorders Identification Test; BDI–Beck Depression Inventory; BYAACQ—Brief Young Adult Alcohol Consequences Questionnaire; CBT-I–Cognitive Behaviour Therapy for Insomnia; DASS 21- Depression, Anxiety and Stress Scale 21 Items; DBAS—Dysfunctional Beliefs About Sleep; DDQ–Daily Drinking Questionnaire; EMA-early morning awakenings; ESS—Epworth Sleepiness Scale; FIRST–Ford Insomnia Response to Stress Test; FIPAQ–Five Item Physical Activity Questionnaire; FSS–Fatigue Severity Scale; GAD-7 –Generalized Anxiety Disorder Scale 7 item; GHQ—General Health Questionnaire; IIS–Insomnia Impact Scale; ISI–Insomnia Severity Index; MOS SLP9 –Medical Outcome Study Sleep Scale; NWAK–Number of Awakenings; PANAS–Positive and Negative Affect Schedule; PHQ—Patient health questionnaire; PMH–Positive Mental Health Scale, PROMIS-SRI SF—Patient Reported Outcomes Measurement Information System—Sleep Related Impairment Short Form; PSS–Perceived Stress Scale; PSWQ—Penn State Worry Questionnaire; PSQI–Pittsburgh Sleep Quality Index; PWS–Psychological Wellbeing Scale; SE–Sleep Efficiency; SCI- Sleep Condition Indicator; SHI–Sleep Hygiene Index; SHPS–Sleep Hygiene Practice Scale;; SOL—Sleep Onset Latency; SPEQ—Specific Psychotic Experiences questionnaire; SQS–Sleep Quality Scale; SRDI–Smith Relaxation Disposition Inventory; SSI- Scale for Suicidal Ideation; STAI-S—State Trait Anxiety Inventory-state; TAP–Test of Attentional Performance; TST–Total Sleep Time; VLMT- Verbal Learning and Memory Test; WASO–Wake After Sleep Onset; WEMWBS—Warwick–Edinburgh Mental Wellbeing Scale; WMS–Wechslser Memory Scale; WSAS—Work and Social Adjustment Scale.

#### Recruitment

Five studies used incentives for participation including course credit (k = 2), cash payments (k = 1) or a prize (k = 2). Other studies recruited participants through voluntary recruitment (k = 6) or advertised the study as offering an intervention that would improve sleep (k = 3) or reduce stress (k = 3). Five studies did not report details on how participants were recruited or incentivised to participate.

#### Comorbidity

The presence of comorbid mental or physical conditions was recorded in seven of the studies. Depression rates at baseline were high in most of the seven studies that reported comorbidity at baseline. Six of the seven studies reported that more than half the participants had depression scores in the clinical range. In one study the presence of suicidal ideation was an inclusion criteria [30]. Rates of anxiety, reported in five studies, were similarly high, with between 41% and 64% of participants having clinical levels of anxiety. In one study, participants had comorbid heavy drinking as the intervention aimed to address both sleep and alcohol consumption [42]. Other studies excluded all participants with comorbid chronic physical illnesses [30, 43, 44], diagnosed insomnia [32], sleep disorders other than insomnia [27, 44], taking medication that could influence sleep [44, 45], taking medication for a psychological disorder or sleep difficulty [32] or students with comorbid or past psychological disorders [27, 29, 30, 32, 44].

#### Intervention

Eight of the 22 studies used a full CBT-I intervention that included psychoeducation, cognitive therapy and behavioural strategies targeting sleep disturbance [1, 27, 29, 42, 45–48]. In another study, cognitive refocusing treatment for insomnia (CRT-I) was used in isolation [49]. This cognitive technique aims to shift thinking away from negative or emotionally arousing topics to a mental activity that is calming, prior to sleep. Two studies [43, 44] used an intervention focused primarily on relaxation techniques. Sleep hygiene was the primary intervention in five studies [36, 50–53]. Six studies focused on mindfulness interventions [28, 30–32, 54, 55] utilising face-to-face sessions [30, 31, 54, 55], videoconferencing [28], or an audio file [32] to give instructions on how to practice mindfulness. All of these mindfulness studies prescribed follow up practice sessions.

#### Delivery format

Just under half of the studies (k = 9) made use of a lecture, workshop, or slide presentation delivered face-to-face [30, 32, 43, 44, 48–50, 54, 55] and one study made use of videoconferencing sessions [28]. Of the remaining studies, ten used some form of online delivery, via email [29, 53], text message [36, 51], or web based program [1, 42, 45–47, 52]. Three studies offered a face-to-face group program [27, 31, 45].

#### Control groups

A non-active control group (waitlist or care as usual) was used in 14 of the studies [1, 27, 28, 30–32, 36, 43, 44, 46, 50, 52, 54, 55]. Of the remaining eight studies with active control groups, four used sleep hygiene as a control group [42, 48, 49, 53], one used sleep monitoring [29], one used a control group including only cognitive therapeutic components [49], one made use of a puzzles task [47] and one made use of a control group offering healthy lifestyle tips [51].

#### Outcome measures

Sleep outcomes were measured by self-report sleep quality questionnaires in all but one of the studies (k = 21), the remaining study only made use of sleep parameters [44]. Sleep parameters (e.g. total sleep time, sleep onset latency) were reported by six studies using self-report sleep diaries (k = 5) or actigraphy (k = 2). None of the studies used polysomnography. Mental health outcomes were included in more than half the studies (k = 12): nine studies reported on depression or anxiety [1, 28, 29, 31, 44, 46, 47, 52, 55], seven reported on stress [28, 30–32, 46, 47, 55] and three studies reported general wellbeing [1, 28, 44].

Very few studies included measures of daytime functioning such as physical activity [28], quality of life, work and social adjustment as outcomes [1, 44]. Two studies reported daytime sleepiness [44, 52] and one on the frequency of alcohol consumption as outcomes [42].

### Meta-analysis

**Fig 2:**
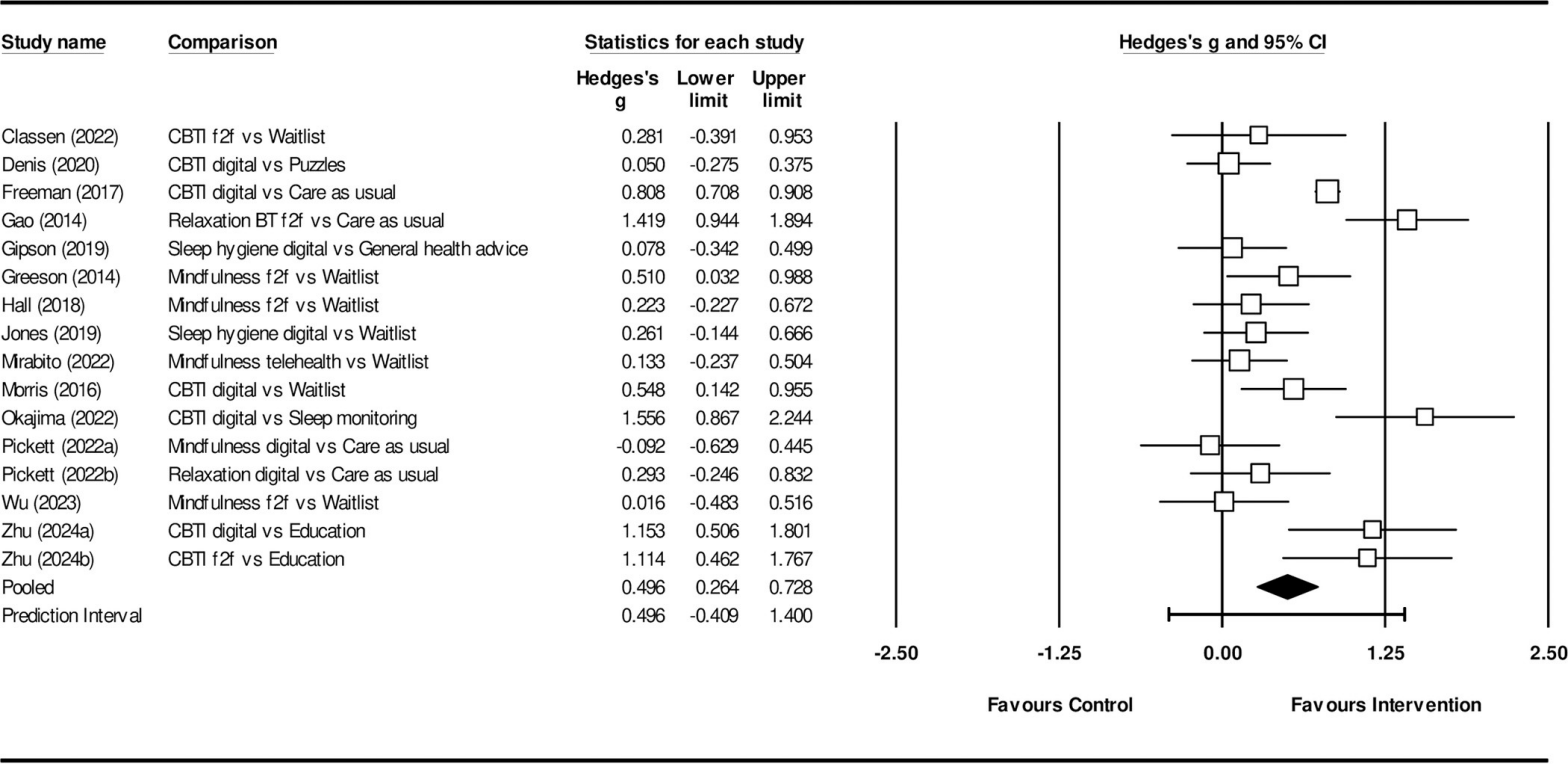
The overall effect of psychological treatment on sleep quality.

Self-reported sleep quality was the only outcome measure with sufficient data for meta-analysis. There were not enough studies reporting on mental health for these variables to be included in the meta-analysis, with only four studies reporting data for depression [1, 28, 29, 46], three studies reporting data on anxiety [28, 29, 47] and four studies reporting stress as an outcome measure [28, 30, 32, 47]. Three of the 22 studies had insufficient data published to include in the meta-analysis [31, 50, 52], four used sleep hygiene as a control group [42, 48, 49, 53] and one did not report sleep quality as an outcome [44]. Therefore, the meta-analysis was based on the remaining 14 studies. The overall between group effect sizes for psychological interventions compared to controls was moderate (n = 14, *g* = 0.50, 95%CI: 0.26–0.73, *I*^*2*^ = 81.75) favouring the intervention groups (Fig 2). Heterogeneity levels were high. Sub-group analyses were used to explore possible sources of this heterogeneity. These analyses included a comparison of the effect of psychological interventions on sleep quality in studies with non-active control groups, such as waitlist or care as usual, vs those with active control groups, such as sleep education, general health advice, or cognitive tasks. The difference between effect sizes was not significant (active control groups: n = 4, *g* = 0.70, 95%CI: 0.25–1.14; non-active control groups: n = 10, *g* = 0.42, 95%CI: 0.13–0.70; Q^*^_bet_ = 4.00, *df* = 1, *p* = 0.05, *I*^*2*^ = 81.75) see S1 Fig in supplementary materials.

### Subgroup analysis

**Fig 3:**
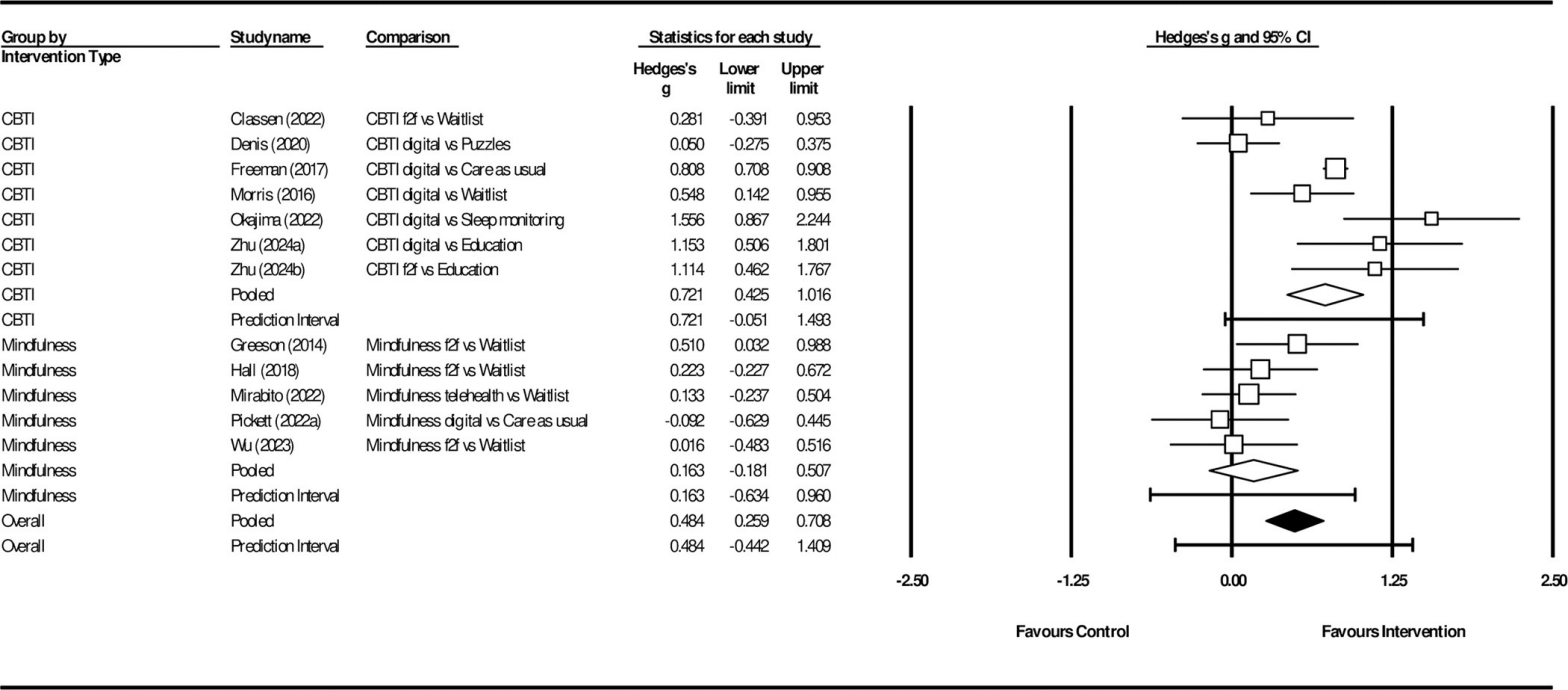
Subgroup analysis: The effect of CBT-I and mindfulness on sleep quality.

A subgroup analysis was conducted to compare the effect size relative to control of studies which focused on mindfulness with CBT-I interventions. Studies were coded into the CBT-I category if they used standard multicomponent CBT-I [1, 27, 29, 45–47]. The mindfulness subgroup included studies that were focused on mindfulness interventions [28, 30, 54, 55]. One study [32] had both mindfulness and relaxation groups compared against a control group, so only the mindfulness group was included under this subgroup. There were significant differences in the effect sizes relative to control between studies that used CBT-I and mindfulness as the intervention (CBT-I: n = 6, *g* = 0.72, 95% CI: 0.43–1.02; mindfulness: n = 5, *g* = 0.16, 95% CI: -0.18–0.51; Q*bet = 26.63, df = 1, p = 0.00, *I*^*2*^ = 81.62), see Fig 3.

### Publication bias

There was no evidence of publication bias observed within the funnel plot, or when conducting Duval and Tweedie’s trim and fill procedure. See S2 Fig in supplementary materials.

### Quality assessments

**Fig 4:**
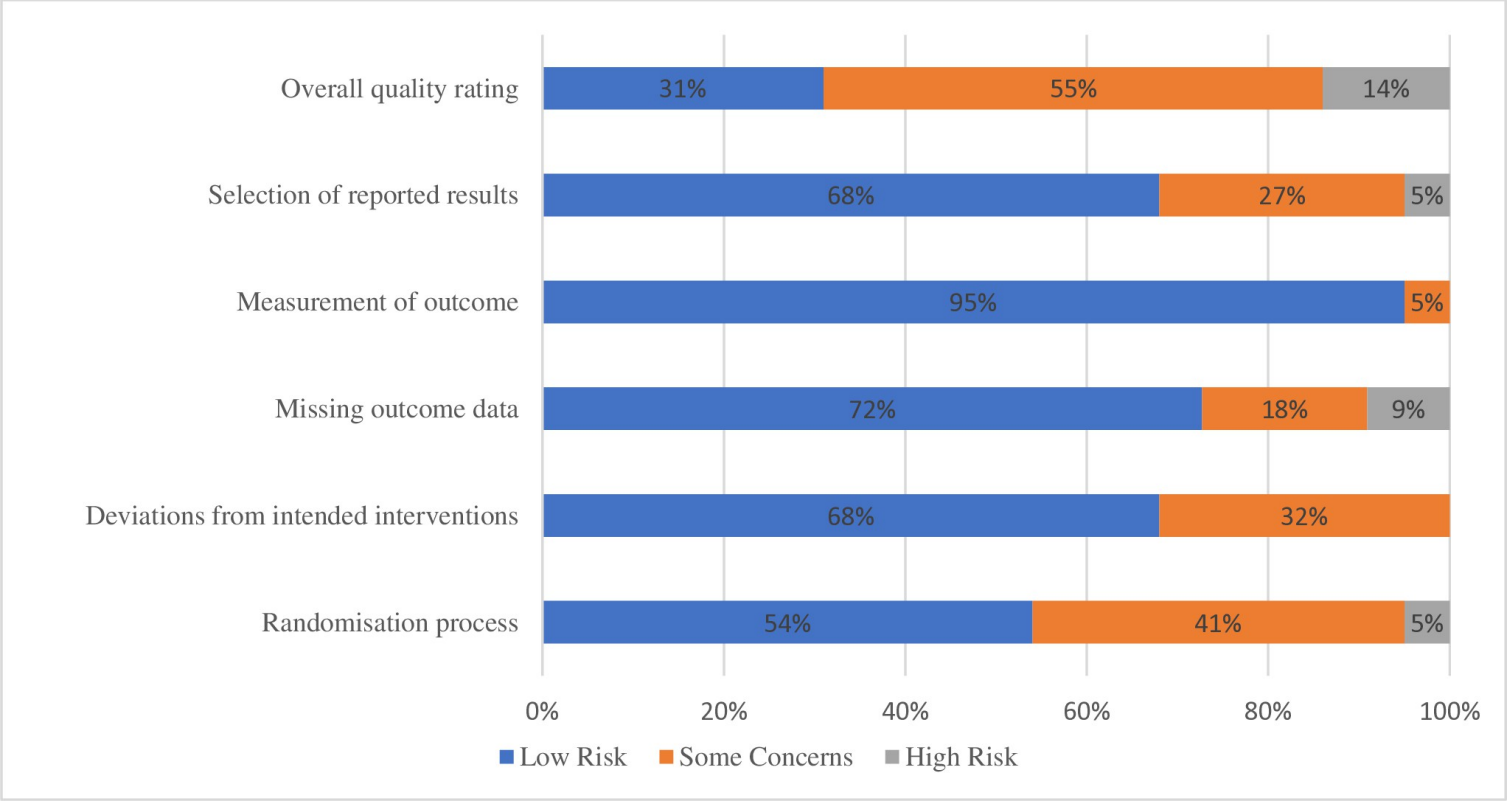
Risk of bias.

The overall methodological quality of studies varied. All reviewers of study quality (MT, AW-S, JN) were consistent in their evaluations of the studies. Overall, 4 (18%) of the studies had a high risk of bias [27, 28, 31, 43]; 12 (55%) of the studies had some concerns over risk of bias [29, 30, 32, 36, 44, 45, 48–51, 53, 55] and 6 (27%) had a low risk of bias [1, 42, 45, 46, 52, 54]. For further details see Fig 4.

## Discussion

This study is a comprehensive review of published randomised controlled trials evaluating psychological treatments for sleep disturbance in university students. Our aim was to examine the impact of psychological interventions on subjective sleep quality and objective sleep indices. We identified 22 RCTs with 6179 participants. Overall, the results of this review indicate that there is emerging evidence that supports the use of psychological interventions as a treatment for sleep disturbance in young adult university students.

This review found three main interventions evaluated in tertiary student populations: CBT-I (36%), sleep hygiene (23%) and mindfulness (27%). Comparators were mostly inactive (64%), however 8 studies (36%) used active control groups, of which, 4 used sleep hygiene. The majority of treatment programs were brief in nature and 18 (82%) were delivered online or as a face-to-face lecture or slide presentation. Only four studies (18%) had face to face sessions of group therapy. Programs were evaluated mainly from self reported sleep quality with few studies (9%) reporting objective sleep parameters. This review was unique in excluding studies with very small sample sizes, including RCTs with both active and non-active control groups and in conducting a comparative subgroup analysis of different types of psychological intervention.

In general, psychological treatments for sleep difficulties were found to improve subjective sleep quality with a moderate effect size (*g* = 0.50), relative to control groups. The effect size found in this review is somewhat smaller than the effect size reported in Saruhanjan et al. [25]: *g* = 0.61. The effect size reported in this previous review [25] may have been inflated due to the inclusion of research studies with small sample sizes. Small trials are associated with larger effect sizes and there is greater heterogeneity between small studies when compared to larger trials [56]. Therefore, the findings of meta-analyses that focus on small trials are more likely to yield imprecise effect size estimates.

The subgroup analysis revealed that effect sizes were significantly different between the intervention types. CBT-I interventions improved sleep quality relative to control with a medium effect size (*g* = 0.72), while mindfulness meditation interventions had a relatively smaller effect size relative to control (*g* = 0.16). The difference could be the result of factors such as the intensity of the intervention, as the CBT-I interventions tended to be longer in duration and are generally multicomponent packages that offer participants a wide range of strategies. In contrast, interventions making use of meditation tended to be much briefer in duration or typically only offered one treatment component, whereas CBT entails multiple treatment components. It is important to note that the subgroup analysis was based on a relatively small number of studies. Further research that specifically compares intervention types are needed to establish the relative efficacy of different interventions.

The capacity of psychological interventions to improve subjective reports of sleep quality in tertiary student populations is supported by this review. However, the existing literature is comprised of a significant number of studies with low participant numbers. Specifically, many studies did not use an intention to treat analytic approach. This may have inflated treatment effects through the automatic exclusion of participants who dropped out of the intervention due to adverse effects or a poor response to treatment. In addition, there was often a lack of clarity in the reporting of randomisation processes. This undermines the validity of the findings reported as in many studies it was unclear if allocation was truly random. Further, sufficiently powered high quality RCTs would strengthen the evidence supporting psychological treatments for sleep quality in this population. In addition, the available research does not often report sleep parameters (such as total sleep time, sleep onset latency, number of awakenings after sleep onset) from either subjective (diaries) or objective (polysomnography, actigraphy) measures. In comparison with subjective self-report measures, objective measures of sleep quality are unbiased by demand characteristics and error that can result from inaccuracy in participant self-reporting. Research that includes both objective and subjective measures of sleep parameters would address this gap in the literature and give a more complete picture of the efficacy of psychological treatments for insomnia.

Future research could also further investigate how key areas of daytime functioning (such as sleepiness, quality of life, academic or work performance, social relationships and health behaviours) are affected by interventions for sleep disturbance. There are few studies currently available that investigate how these variables are affected by treatment. The inclusion of these outcome variables would provide a greater understanding of the broader potential benefits of addressing untreated sleep disturbance [33]. It is also unclear what precise mechanisms are involved in the improvement of sleep following psychological treatment. Given that many treatment approaches, such as CBT-I, are multicomponent treatment packages, the mechanism by which sleep improves and what components of treatment are the most active is not yet clear [57, 58]. A systematic review of potential mediators of CBT-I found some evidence indicating reductions in dysfunctional beliefs about sleep, and to a lesser extent, reductions in pre-sleep hyperarousal are potential mediators of improvements in sleep quality however, as the authors note, further research is needed to verify their findings [59].

### Limitations

There were high levels of heterogeneity found in this review, which suggest the overall effect size should be interpreted with caution. It is likely that the cause of this heterogeneity was the use of studies that were investigating different types of treatments (CBT-I, mindfulness, relaxation, sleep hygiene) being delivered in different modalities (face to face, digital, group) and with differing treatment durations and levels of intensity. Therefore, the conclusions drawn from these studies should be interpreted cautiously. Furthermore, this review was limited in scope by focusing exclusively on psychological interventions for sleep disturbance. This excluded a range of non-pharmacological interventions for insomnia that could be beneficial, such as physical activity, the use of music, and light therapy to manage circadian rhythms.

Another limitation is that study samples were primarily non treatment seeking, general student samples. About half the studies (55%) had no sleep disturbance symptom criteria for entry and only 10% of studies required a full insomnia diagnosis for study entry. Consequently, it is likely that the overall sample from the combined studies had low baseline sleep disturbance symptom severity. The findings of this review may not be generalisable to university students with greater sleep difficulties, or those that meet full insomnia criteria. That said, to see effect sizes of this magnitude in a sample with low symptom levels to begin with suggests that these interventions, if delivered at scale to this same population, could have significant public health benefits. Nonetheless, further research with university students experiencing clinical levels of insomnia would determine whether psychological interventions are suitable for both subclinical and clinical populations.

### Summary

In summary, this systematic review of psychological treatments designed to improve sleep quality in university students provides an updated synthesis of RCTs. There is evidence from this review that psychological treatments improve sleep quality in young adult university students. These findings are consistent with the sleep disturbance literature with adults in general and highlight the value of psychological treatments to improve sleep. Furthermore, such interventions may offer a way to indirectly improve the mental health of university students which is not associated with the same level of stigma as other mental health problems [60]. Further research is needed to explore how psychological treatments affect objective measures of sleep quality and daytime functioning. The optimal type of psychological intervention to improve sleep quality in university students would also be a useful focus of future research. Although the largest effect sizes were observed with CBT-I interventions in this review, further research is needed to determine the utility of this approach with young adult university students.

## Supporting information

S1 Checklist PRISMA checklist. (DOCX)

S1 Fig Control subgroup type subgroup analysis. (TIF)

S2 Fig Publication bias funnel plot. (TIF)

S1 File Search Strategy. (DOCX)

S1 Table Exclusion reasons for article selection. (DOCX)

S2 Table Data for meta-analysis. (XLSX)

S3 Table Risk of bias details. (XLSX)

## References

[1] Freeman D, Sheaves B, Goodwin GM, Yu LM, Nickless A, Harrison PJ, et al. The effects of improving sleep on mental health (OASIS): a randomised controlled trial with mediation analysis. Lancet Psychiatry. 2017;4: 749–758. doi: 10.1016/S2215-0366(17)30328-0 28888927 PMC5614772

[2] Baglioni C, Battagliese G, Feige B, Spiegelhalder K, Nissen C, Voderholzer U, et al. Insomnia as a predictor of depression: A meta-analytic evaluation of longitudinal epidemiological studies. J Affect Disord. 2011;135: 10–19. doi: 10.1016/j.jad.2011.01.011 21300408

[3] Pigeon WR, Bishop TM, Krueger KM. Insomnia as a Precipitating Factor in New Onset Mental Illness: A Systematic Review of Recent Findings. Curr Psychiatry Rep. 2017;19: 1–11. 28616860 10.1007/s11920-017-0802-x

[4] Lovato N, Gradisar M. A meta-analysis and model of the relationship between sleep and depression in adolescents: Recommendations for future research and clinical practice. Sleep Med Rev. 2014;18: 521–529. doi: 10.1016/j.smrv.2014.03.006 24857255

[5] Irwin MR, Carrillo C, Sadeghi N, Bjurstrom MF, Breen EC, Olmstead R. Prevention of Incident and Recurrent Major Depression in Older Adults With Insomnia: A Randomized Clinical Trial. JAMA Psychiatry. 2022;79: 33–41. doi: 10.1001/jamapsychiatry.2021.3422 34817561 PMC8733847

[6] Bei B, Asarnow LD, Krystal A, Edinger JD, Buysse DJ, Manber R. Treating Insomnia in Depression: Insomnia Related Factors Predict Long-Term Depression Trajectories. J Consult Clin Psychol. 2018;86: 282–293. doi: 10.1037/ccp0000282 29504795 PMC5841584

[7] Buysse DJ, Tu XC, Cherry CR, Begley A, Kowalski J, Kupfer DJ, et al. Pretreatment REM Sleep and Subjective Sleep Quality Distinguish Depressed Psychotherapy Remitters and Nonremitters. Biol Psychiatry. 1999;45: 205–213. doi: 10.1016/s0006-3223(98)00198-x 9951568

[8] Meaklim H, Jackson ML, Bartlett D, Saini B, Falloon K, Junge M, et al. Sleep education for healthcare providers: Addressing deficient sleep in Australia and New Zealand. Sleep Health. 2020;6: 636–650. doi: 10.1016/j.sleh.2020.01.012 32423774

[9] Dzierzewski JM, Griffin SC, Ravyts S, Rybarczyk B. Psychological Interventions for Late-Life Insomnia: Current and Emerging Science. Sleep Aging. 2018;4: 268–277. doi: 10.1007/s40675-018-0129-0 31106115 PMC6519733

[10] Morin C, Bootzin RR, Buysse DJ, Edinger JD, Espie CA, Lichstein KL. Psychological And Behavioral Treatment Of Insomnia:Update Of The Recent Evidence (1998–2004. Sleep. 2006;29: 1398–1414. doi: 10.1093/sleep/29.11.1398 17162986

[11] Van Straten A, Zweerde T, Kleiboer A, Cuijpers P, Morin C, Lancee J. Cognitive and behavioral therapies in the treatment of insomnia: A meta-analysis. Sleep Med Rev. 2018;38: 3–16. doi: 10.1016/j.smrv.2017.02.001 28392168

[12] Mitchell MD, Gehrman P, Perlis M, Umscheid CA. Comparative effectiveness of cognitive behavioral treatment for insomnia: A systematic review. BMC Fam Pr. 2012;13: 1–11. 10.1186/1471-2296-13-40 PMC3481424 22631616

[13] Ree M, Junge M, Cunnington D. Australasian Sleep Association position statement regarding the use of psychological/behavioral treatments in the management of insomnia in adults. Sleep Med. 2017;36: S43–S47. doi: 10.1016/j.sleep.2017.03.017 28648226

[14] Friedrich A, Schlarb A. Let’s talk about sleep: a systematic review of psychological interventions to improve sleep in University students. J Sleep Res. 2018;27: 4–22. 28618185 10.1111/jsr.12568

[15] Foulkes L, McMillan D, Gregory AM. A bad night’s sleep on campus: an interview study of first-year university students with poor sleep quality. Sleep Health. 2019;5: 280–287. doi: 10.1016/j.sleh.2019.01.003 31208711

[16] Lund HG, Reider BD, Whiting AB, Prichard JR. Sleep Patterns and Predictors of Disturbed Sleep in a Large Population of University Students. J Adolesc Health. 2010;46: 124–132. 20113918 10.1016/j.jadohealth.2009.06.016

[17] O’hayon M. Epidemiology of insomnia: what we know and what we still need to learn. Sleep Med Rev. 2002;6: 97–111. doi: 10.1053/smrv.2002.0186 12531146

[18] Jiang X, Zheng X, Yang J, Ye C, Chen Y, Zhang Z, et al. A systematic review of studies on the prevalence of Insomnia in University students. Public Health. 2015;129: 1579–1584. doi: 10.1016/j.puhe.2015.07.030 26298588

[19] Taylor DJ, Bramoweth AD, Grieser EA, Tatum JI, Roane B. Epidemiology of Insomnia in University Students: Relationship With Mental Health, Quality of Life, and Substance Use Difficulties. Behav Ther. 2013;44: 339–348. 23768662 10.1016/j.beth.2012.12.001

[20] Auerbach RP, Mortier P, Bruffaerts R, Alonso J, Benjet C, Cuijpers P, et al. WHO World Mental Health Surveys International College Student Project: Prevalence and distribution of mental disorders. J Abnorm Psychol. 2018;127: 623–638. doi: 10.1037/abn0000362 30211576 PMC6193834

[21] Lipson SK, Lattie EG, Eisenberg D. Increased Rates of Mental Health Service Utilization by U.S. College Students: 10-Year Population-Level Trends (2007–2017. Psychiatr Serv. 2019;70: 60–63. doi: 10.1176/appi.ps.201800332 30394183 PMC6408297

[22] Pigeon WR, Hegel M, Unutzer J, Ming-Yu F, Sateia MJ, Lyness JM, et al. Is Insomnia a Perpetuating Factor for Late-Life Depression in the IMPACT Cohort? Sleep. 2008;31. doi: 10.1093/sleep/31.4.481 18457235 PMC2279755

[23] Dombrovski AY, Cyranowski JM, Mulsant BH, Houck PR, Buysse DJ, Andreescu C, et al. Which symptoms predict recurrence of depression in women treated with maintenance interpersonal psychotherapy? Depress. Anxiety. 2008;25: 1060–1066. 18781665 10.1002/da.20467 PMC2705944

[24] Kodsi A, Bullock B, Kennedy G, Tirlea. Psychological Interventions to Improve Sleep in Young Adults: A Systematic Review and Meta-analysis of Randomized Controlled Tirals. Behav Sleep Med. 2022;20: 125–142. doi: 10.1080/15402002.2021.1876062 33554644

[25] Saruhanjan K, Zarski A, Baumeiser H, Cuijpers P, Spiegelhalder K, Auerbach RP, et al. Psychological interventions to improve sleep in University students: A meta-analysis of randomized controlled trials. J Sleep Res. 2020; 1–22. 10.1111/jsr.13097 32672865

[26] Mohr DC, Spring B, Freedland KE, Beckner V, Arean P, Hollon SD, et al. The Selection and Design of Control Conditions for Randomized Controlled Trials of Psychological Interventions. Psychother Psychosom. 2009;78: 275–284. doi: 10.1159/000228248 19602916

[27] Classen M, Friedrich A, Schlarb AA. Sleep better–Think better!–The effect of CBT-I and HT-I on sleep and subjective and objective neurocognitive performance in university students with insomnia. Cogent Psychol. 2022;9. doi: 10.1080/23311908.2022.2045051

[28] Mirabito G, Verhaeghen P. Remote delivery of a Koru Mindfulness intervention for college students during the COVID-19 pandemic. J Am Coll Health. 2022. doi: 10.1080/07448481.2022.2060708 35427456

[29] Okajima I, Tanizawa N, Harata M, Su S, Yang C, LI X, et al. Can an E-Mail-Delivered CBT for Insomnia Validated in the West Be Effective in the East? A Randomized Controlled Trial. Int J Environ Health Res Public Health. 2022;19: 186. doi: 10.3390/ijerph19010186 35010445 PMC8751173

[30] Wu R, Zhong S, Wang G, Wu M, Xu J, Zhu H, et al. The Effect of Brief Mindfulness Meditation on Suicidal Ideation. Stress Sleep Qual Arch Suicide Res. 2023;27: 215–230,. doi: 10.1080/13811118.2021.1982800 34612785

[31] Gallo GG, Curado DF, Machado MPA, Espíndola MI, Scattone VV, Noto AR. A randomized controlled trial of mindfulness: effects on university students’ mental health. Int J Ment Health Syst. 2023;17: 32. doi: 10.1186/s13033-023-00604-8 37833796 PMC10571349

[32] Pickett SM, Kozak AT, Lanni DJ, Warnke AS, Gaillard P, Jarrett NL. The comparison of brief, online mindfulness and relaxation interventions to reduce stress and improve sleep-related outcomes in college students. J Am Coll Health. 2022. doi: 10.1080/07448481.2022.2066979 35709245

[33] Benz F, Knoop T, Ballesio A, Bacaro V, Johann AF, Rucker G, et al. The efficacy of cognitive and behavior therapies for insomnia on daytime symptoms: A systematic review and network meta-analysis. Clin Psychol Rev. 2020;80: 1–24. doi: 10.1016/j.cpr.2020.101873 32777632

[34] Szucs D, Ioannidis JP. Empirical assessment of published effect sizes and power in the recent cognitive neuroscience and psychology literature. PLOS Biol. 2017;15: 1–18. doi: 10.1371/journal.pbio.2000797 28253258 PMC5333800

[35] Higgins JPT, Savovic J, Page MJ, Elbers RG, Sterne JAC. Assessing risk of bias in a randomized trial Chapter 8. Higgins JPT, Thomas J, Chandler J, Cumpston M, Li T, Page M, et al., editors. Cochrane Handb Syst Rev Interv Second Ed. 2019; 205–228.

[36] Jones KE, Evans R, Forbes L, Shoenberger Y, Heaton K, Snyder S. Research on freshman and sleeping habits: A text message-based sleep intervention. Journal of American University Health; 2019. 10.1080/07448481.2019.1626860 31210596

[37] Cohen J. Statistical power analysis for behavioral sciences. Erlbaum; 1988.

[38] Borenstein M, Hedges LV, Higgins JPT, Rothstein HR. Introduction to meta-analysis. Wiley; 2009.

[39] Egger M, Davey Smith G, Schneider M, Minder C. Bias in meta-analysis detected by a simple, graphical test. BMJ. 1997;315: 629–634. doi: 10.1136/bmj.315.7109.629 9310563 PMC2127453

[40] Duval S, Tweedie R. A Nonparametric “Trim and Fill” Method of Accounting for Publication Bias in Meta-Analysis. J Am Stat Assoc. 2000;95: 89–98. doi: 10.1080/01621459.2000.10473905

[41] Duval S, Tweedie R. Trim and Fill: A Simple Funnel-Plot-Based Method of Testing and Adjusting for Publication Bias in Meta-Analysis. Biometrics. 2000;56: 455–463. doi: 10.1111/j.0006-341x.2000.00455.x 10877304

[42] Fucito LM, DeMartini KS, Hanrahan TH, Yaggi HK, Heffern C, Redeker NS. Using Sleep Interventions to Engage and Treat Heavy-Drinking University Students: A Randomized Pilot Study. Alcohol Clin Exp Res. 2017;41: 798–809. 28118486 10.1111/acer.13342 PMC5378596

[43] Gao R, Lv Y, Li X, Zhou K, Jin X, Dang S, et al. Effects of comprehensive sleep management on sleep quality in University students in mainland China. Sleep Biol Rhythms. 2014;12: 194–202.

[44] Means MK, Lichstein KL, Epperson MT, Johnson CT. Relaxation therapy for insomnia: Nighttime and day time effects. Behav Res Ther. 2000;38: 665–678. doi: 10.1016/s0005-7967(99)00091-1 10875189

[45] Zhu K, Xue S. Effect of cognitive behavioral therapy for insomnia on sleep quality among college students: the role of hyperarousal and dysfunctional beliefs. Behav Sleep Med. 2024; 1–15. doi: 10.1080/15402002.2024.2401473 39267307

[46] Morris J, Firkins A, Millings A, Mohr C, Redford P, Rowe A. Internet-delivered cognitive behavior therapy for anxiety and insomnia in a higher education context. Anxiety Stress Coping Int J. 2016;29: 415–431. 10.1080/10615806.2015.1058924 26079158

[47] Denis D, Eley TC, Rijsdijk F, Zavos HMS, Keers R, Espie CA, et al. Is digital cognitive behavioural therapy for insomnia effective in treating sub-threshold insomnia: a pilot RCT. Sleep Med. 2020;66: 174–183. doi: 10.1016/j.sleep.2019.10.007 31901759

[48] Kloss JD, Nash CO, Walsh CM, Culnan E, Horsey S, Sexton-Radek K. A “Sleep 101” Program for University Students Improves Sleep Hygiene Knowledge and Reduces Maladaptive Beliefs about Sleep. Behav Med. 2016;42: 48–56. 25268924 10.1080/08964289.2014.969186

[49] Gellis LA, Arigo D, Elliott JC. Cognitive Refocusing Treatment for Insomnia: A Randomized Controlled Trial in University Students. Behav Ther. 2013;44: 100–110. doi: 10.1016/j.beth.2012.07.004 23312430

[50] Barber LK, Cucalon MS. Modifying the Sleep Treatment Education Program for Students to include technology use (STEPS-TECH): Intervention effects on objective and subjective sleep outcomes. Stress Health. 2017;33: 684–690. doi: 10.1002/smi.2746 28156049

[51] Gipson CS, Chilton JM, Dickerson SS, Alfred D, Haas BK. Effects of a sleep hygiene text message intervention on sleep in University students. J Am Coll Health. 2019;67: 32–41. 29652630 10.1080/07448481.2018.1462816

[52] Hershner S, O’Brien LM. The Impact of a Randomized Sleep Education Intervention for University Students. J Clin Sleep Med. 2018;14: 337–347. doi: 10.5664/jcsm.6974 29510791 PMC5837835

[53] Mairs L, Mullan B. Self-Monitoring vs. Implementation Intentions: a Comparison of Behaviour Change Techniques to Improve Sleep Hygiene and Sleep Outcomes in Students. Int J Behav Med. 2015;22: 635–644. doi: 10.1007/s12529-015-9467-1 25673110

[54] Greeson JM, Juberg MK, Maytan M, James K, Rogers H. A randomized controlled trial of Koru: a mindfulness program for University students and other emerging adults. J Am Univ Health J ACH. 2014;62: 222–233. 10.1080/07448481.2014.887571 PMC4016159 24499130

[55] Hall BJ, Xiong P, Guo X, Sou EKL, Chou UI, Shen Z. An evaluation of a low intensity mHealth enhanced mindfulness intervention for Chinese University students: A randomized controlled trial. Psychiatry Res. 2018;270: 394–403. doi: 10.1016/j.psychres.2018.09.060 30300870

[56] IntHout J, Ioannidis JPA, Borm GF, Goeman JJ. Small studies are more heterogeneous than large ones: a meta-meta-analysis. J Clin Epidemiol. 2015;68: 860–869. doi: 10.1016/j.jclinepi.2015.03.017 25959635

[57] Harvey AG, Bélanger L, Talbot L, Eidelman P, Beaulieu-Bonneau S, Fortier-Brochu É, et al. Comparative efficacy of behavior therapy, cognitive therapy, and cognitive behavior therapy for chronic insomnia: A randomized controlled trial. J Consult Clin Psychol. 2014;82: 670–683. doi: 10.1037/a0036606 24865869 PMC4185428

[58] Morin C, Bei B, Bjorvatn B, Poyares D, Spiegelhalder K, WIng YK. World sleep society international sleep medicine guidelines position statement endorsement of “behavioral and psychological treatments for chronic insomnia disorder in adults: An American Academy of sleep medicine clinical practice guidelines.” Sleep Medicine. 2023;109: 164–169. doi: 10.1016/j.sleep.2023.07.001 37454606

[59] Parsons CE, Zachariae R, Landberger C, Young KS. How does cognitive behavioural therapy for insomnia work? A systematic review and meta-analysis of mediators of change. Clin Psychol Rev. 2021;86: 102027. doi: 10.1016/j.cpr.2021.102027 33887655

[60] Tadros M, Li S, Upton E, Newby J, Werner-Seidler A. Preferences of University Students for a Psychological Intervention Designed to Improve Sleep: Focus Group Study. J Med Internet Res Hum Factors. 2023;10: 44145. doi: 10.2196/44145 37616036 PMC10485721

