# Supplementary figures and images for: A systematic review and meta-analysis of psychological treatments to improve sleep quality in university students
Source: PLoS One. 2025 Feb 13;20(2):e0317125. doi: 10.1371/journal.pone.0317125 (PMC11824969; doi:10.1371/journal.pone.0317125)

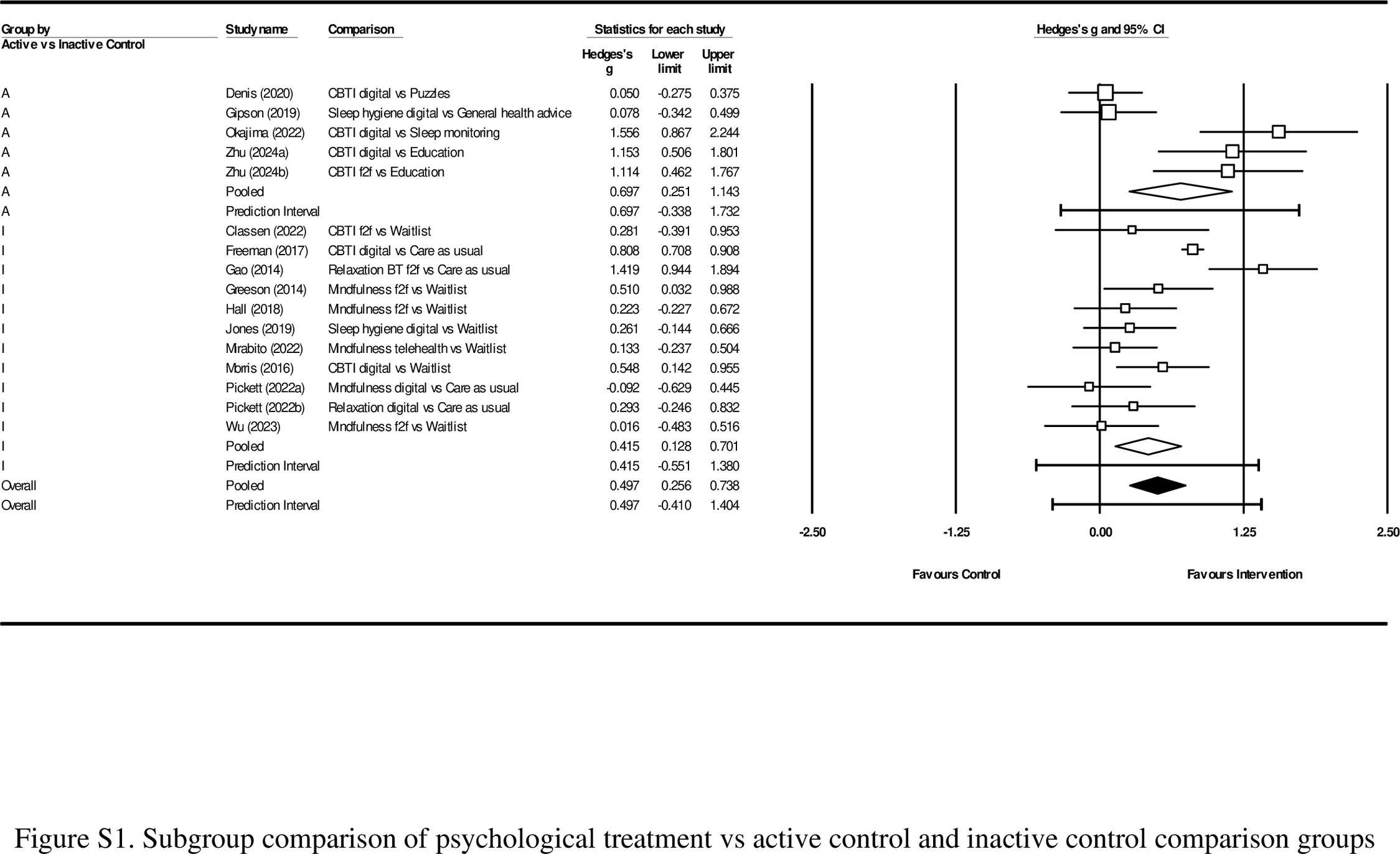

Supplement: S1 Fig — (TIF) [file pone.0317125.s002.tif]

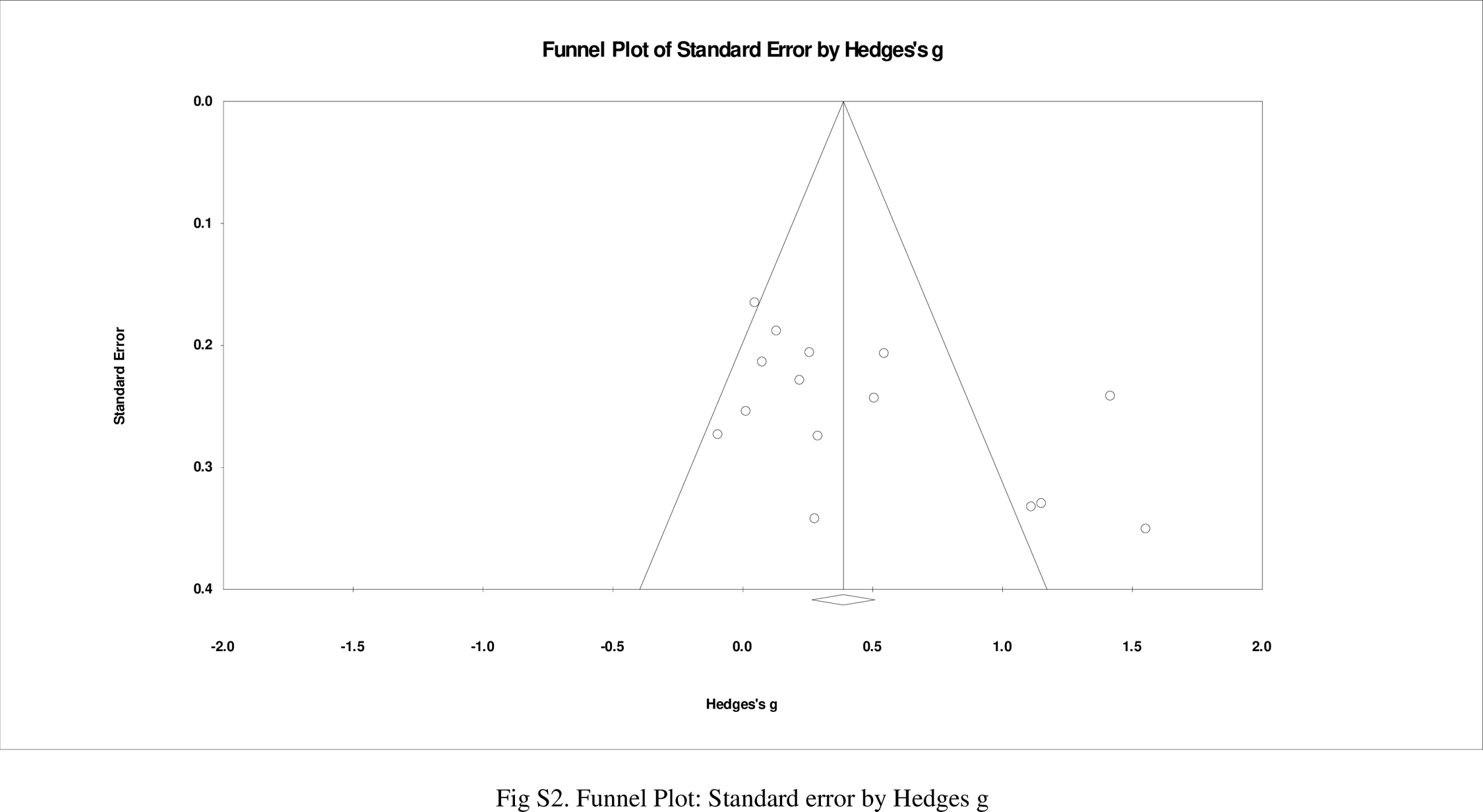

Supplement: S2 Fig — (TIF) [file pone.0317125.s003.tif]
