# Supplementary material for: A systematic review and meta-analysis of psychological treatments to improve sleep quality in university students
Source: PLoS One. 2025 Feb 13;20(2):e0317125. doi: 10.1371/journal.pone.0317125 (PMC11824969; doi:10.1371/journal.pone.0317125)
Supplement: S1 File — (DOCX) [file pone.0317125.s004.docx]

**S3_ file - Supplementary Materials – Search Strategy**

***Topic:*** Psychological Treatments to Improve Sleep Quality in University Students

***Databases:***

EMBASE, PubMed, PsychInfo, Medline search strategy

***Search Date:*** 11 August 2023 update: 20 September 2024

***Languages:*** English

**Strategy for search terms:**

| Terms Connected by **OR** | **AND** | Terms connected by **OR** | **AND** | Terms connected by **OR** |
| --- | --- | --- | --- | --- |
| Sleep related terms |  | Treatment related terms |  | Population terms |
|  |  |  |  |  |

In PubMed and Medline – “all fields” were searched

In Psychinfo and EMBASE – “keywords’ were searched

**Terms for search**

***Sleep related terms***

1. Sleep
2. Sleep Arousal Disorder
3. Sleep Disorders
4. Sleep Wake Disorders
5. Sleep Disorders Intrinsic
6. Sleep Hygiene
7. Sleep Latency
8. Sleep phase chronotherapy
9. Sleep initiation and maintenance disorders
10. Insomnia
11. Sleep Difficulty
12. Sleep disturbance
13. Dreams
14. Nightmare
15. Night terrors
16. Nightmare disorder

***Treatment related terms***

1. Behav* Therapy
2. Behav* Treatment
3. Behav* intervention
4. Treatment
5. Therapeutics
6. Cognitive behav* therapy
7. Cognitive therapy
8. Cognitive intervention
9. Distraction
10. Meditation
11. Mindfulness
12. Metacognit*
13. Acceptance commitment therapy
14. Self compassion

***Population terms***

1. Young adults
2. Youth
3. University students
4. Undergraduate students
5. Postgraduate students
6. College students
7. Tertiary students
8. Young people
9. Freshman
