# Supplementary material for: A systematic review and meta-analysis of psychological treatments to improve sleep quality in university students
Source: PLoS One. 2025 Feb 13;20(2):e0317125. doi: 10.1371/journal.pone.0317125 (PMC11824969; doi:10.1371/journal.pone.0317125)
Supplement: S1 Table — (DOCX) [file pone.0317125.s005.docx]

| Number | Authors | Include/Exclude | Reason for Exclusion - notes from MT / SL / AWS | Date of Data extraction |
| --- | --- | --- | --- | --- |
| 1 | Chang et al 2021 | Exclude | exclude no randomisation | NA |
| 2 | Miller 2020 | Exclude | Study involves non tertiary sample | NA |
| 3 | Duraccio et al 2021 | Exclude | Exclude not a psych intervention | NA |
| 4 | Faaland et al 2023 | Exclude | Exclude population criteria not met | NA |
| 5 | Goodhines et al 2022 | Exclude | exclude less than 20 per group | NA |
| 6 | Ma et al 2023 | Exclude | exclude the control does not control for psychological aspects of the intervention | NA |
| 7 | Victor 2019 | Exclude | Non-randomised quasi exp study testing relaxation in university sample with primary insomnia | NA |
| 8 | Zwart 1979 | Exclude | Compared stimulus control to other conditions and looked at sleep parameters | NA |
| 9 | Store et al 2022 | Exclude | exclude not teriary sample | NA |
| 10 | Ubarra et al 2022 | Exclude | exclude same data set as Okajima | NA |
| 11 | Trockel 2011 | Exclude | Non-randomised trial comparing email delivered CBT-I vs a mood intervention in college kids | NA |
| 12 | Tsai 2004 | Exclude | Non-randomised comparison between a uni course on sleep management vs nothing, no age reported but all students | NA |
| 13 | Thakre 2024 | Exclude | not clear that participants were randomized | NA |
| 14 | Schlarb 2017 | Exclude | Pre-post design evaluating CBT-I + Hypnotherapy for uni students with sleep problems | NA |
| 15 | Shealy 1979 | Exclude | Experimental study comparing three interventions and two control groups for sleep disturbance | NA |
| 16 | Taylor 2014 | Exclude | Pilot evalution of CBT-I in uni students (apparently first randomised test of CBT-I in this sample) | NA |
| 17 | Aritake-Okada 2009 | Exclude | Not a tertiary sample | NA |
| 18 | Bedford 2018 | Exclude | Veteran sample, not a tertiary sample | NA |
| 19 | Bonin 2014 | Exclude | Not a tertiary sample | NA |
| 20 | Bostock 2016 | Exclude | Not a tertiary sample | NA |
| 21 | Kaplan 2018 | Exclude | Not a tertiary sample sample have bipolar disorder | NA |
| 22 | Mooney 2009 | Exclude | Not a tertiary sample (M=32.89) | NA |
| 23 | Yang 2018 | Exclude | Not psychological intervention | NA |
| 24 | Friedrich 2018 | Exclude | Meets all criteria but did not report sleep outcomes (only MH outcomes) | NA |
| 25 | Quan 2013 | Exclude | The outcome is sleep knowledge, not actual sleep quality/duration/improvement (not behavioural measurement) | NA |
| 26 | Todd 2014 | Exclude | Primary outcomes are sleep hygiene not sleep duration/quality etc | NA |
| 27 | Ye 2014 | Exclude | Focuses on sleep knowledge, not actual sleep. Designed for education not for improvement of participants sleep. | NA |
| 28 | Arzilli 2019 | Exclude | Not a treatment for insomnia/sleep difficulties, participants with sleep problems excluded | NA |
| 29 | Ball 2002 | Exclude | Multi-health component intervention, does not include sleep as a primary or key outcome (Epworth included as one of >20 outcomes) | NA |
| 30 | Brown 2010 | Exclude | Does not involve an intervention | NA |
| 31 | Cordi 2014 | Exclude | Uses a hypnosis intervention to improve slow wave sleep (this is borderline - could be a part of sleep disturbance but not insomnia) | NA |
| 32 | Guestella 2007 | Exclude | Experimental study, does not meet intervention criteria | NA |
| 33 | Kloss 2011 | Exclude | Review article | NA |
| 34 | Kor 2011 | Exclude | Does not test an intervention | NA |
| 35 | Kushner 2011 | Exclude | Participants chose which behaviour to target, not standardied, no sleep intervention | NA |
| 36 | Mak 2017 | Exclude | Interventions generally targetting mental health, not sleep specifically | NA |
| 37 | Moore 2012 | exclude | Not a sleep-focused intervention (multiple health) and no sleep outcome | NA |
| 38 | Peltz 2016 | Exclude | Cross sectional study, does not involve an intervention | NA |
| 39 | Valshtein 2020 | Exclude | Targets bedtime procrastination, not sleep parameters (but does include sleep parameters as outcomes). Could go either way on this one - I think this intervention is novel and could be used in combination with CBT-I to facilitate behavoiur change | NA |
| 40 | Werch 2008 | Exclude | Multiple component intervention not specifically focused on sleep | NA |
| 41 | Woods 2009 | Exclude | Looks of the role of selective attention in sleep using attn bias task. Not a psychological intervention designed to improve sleep. | NA |
| 42 | Baehr 2003 | Exclude | Not a psychological intervention (nocturnal exercise) | NA |
| 43 | Caldwell 2009 | Exclude | Uses physical interventioons - pilates and Tajiquan (martial arts) | NA |
| 44 | Caldwell 2011 | Exclude | Uses Tajiquan (looks at mindfulness as a mediator) but still looks mostly like a physical intervention | NA |
| 45 | Caldwell 2016 | Exclude | Uses Tai Chi Chuan - physical intervention | NA |
| 46 | Chen 2014 | Exclude | Not a psychological intervention (music) | NA |
| 47 | Danielsoon 2016 | Exclude | Light therapy + CBT vs Light therapy. Inclined to exclude, no standalone psychological intervention tested | NA |
| 48 | Danielsson 2018 | Exclude | Used light therapy not a psychological intervention in DPSD sample | NA |
| 49 | Esaki 2017 | Exclude | Not a psychological intervention | NA |
| 50 | Forquer 2007 | Exclude | Not a psychological intervention (white noise) | NA |
| 51 | Laszlo 2007 | Exclude | Music intervention | NA |
| 52 | Lazic 2007 | Exclude | Music intervention | NA |
| 53 | Oxtoby 2013 | Exclude | Music intervention | NA |
| 54 | Saxvig 2013 | Exclude | Intervention not psychological (bright light and melatonin) | NA |
| 55 | Trahan 2018 | Exclude | Music intervention, mixed methods analysis | NA |
| 56 | Tsai 2015 | Exclude | Not a psychological intervention (paced breathing) | NA |
| 57 | Zheng 2015 | Exclude | RCT testing Tai Chi. Not a psychological intervention, no sleep outcomes | NA |
| 58 | Abramowitz 2008 | Exclude | PTSD sample - not representative of the general population | NA |
| 59 | Arora 2007 | Exclude | Medical interns not representative of the general population | NA |
| 60 | Block 2010 | Exclude | Schizophrenic sample - not representative | NA |
| 61 | Caia 2018 | Exclude | Sample not representative of the general population (athletes) | NA |
| 62 | Epstein 2013 | Exclude | Veteran sample | NA |
| 63 | Fillon 2015 | Exclude | Borderline - sample not representative (treatment seeking for smoking cessation - likely to differ on key characteristics to general population) | NA |
| 64 | Park 2020 | Exclude | Traumatised refugee sample | NA |
| 65 | Randall 2019 | Exclude | Prison sample | NA |
| 66 | Smitherman 2016 | Exclude | Non-representative sample of migraine sufferers | NA |
| 67 | Vuletic 2016 | Exclude | Participants were current army service members | NA |
| 68 | Wagley 2012 | Exclude | Participants not representative of general population - psychiatric outpatients, also older | NA |
| 69 | Alvarez 1992 | Exclude | Case series | NA |
| 70 | Asano 2015 | Exclude | No randomisation – quasi experimental design | NA |
| 71 | Borkovec 1973 | Exclude | Study sample too small | NA |
| 72 | Borkovec 1975 | Exclude | Study sample too small | NA |
| 73 | Borkovec 1976 | Exclude | Study sample too small | NA |
| 74 | Borkovec 1978 | Exclude | Study sample too small | NA |
| 75 | Borkovec 1979 | Exclude | Study sample too small | NA |
| 76 | Bradley 2018 | Exclude | Case series | NA |
| 77 | Carney 2006 | Exclude | participant number too small | NA |
| 78 | Carrera 1980 | Exclude | participant number too small | NA |
| 79 | Chu 2018 | Exclude | No control group (both groups got the intervention, one with intervention + wearables) | NA |
| 80 | Digdon 2011 | Exclude | Compared three psych interventions delivered by email to college students | NA |
| 81 | Funderburk 2015 | Exclude | Written up as a retrospective case series | NA |
| 82 | Gershman 1974 | Exclude | Did not include a control group, only a comparison intervention. | NA |
| 83 | Goodie 2009 | Exclude | Case series | NA |
| 84 | Haynes 1974 | Exclude | Study numbers too small | NA |
| 85 | Kahn 1968 | Exclude | Essentially a case series without standardised assessment (but not explicitly stated) | NA |
| 86 | Petrov 2014 | Exclude | *Single group pre-post study testing a one-session behavioural intervention | NA |
| 87 | Zhao 2019 | Exclude | Fitness intervention | NA |
| 88 | Zeng 2015 | Exclude | Tai chi intervention | NA |
| 89 | Mirabito et al 2022 | Include |  | November 2023 (MT checked by AWS/JN) |
| 90 | Pickett et al 2022 | Include |  | November 2023 (MT checked by AWS/JN) |
| 91 | Wu et al 2023 | Include |  | November 2023 (MT checked by AWS/JN) |
| 92 | Okajima 2022 | Include |  | November 2023 (MT checked by AWS/JN) |
| 93 | Gallo 2023 | Include |  | September 2024 (MT checked by SL) |
| 94 | Denis 2020 | Include |  | September 2024 (MT checked by SL) |
| 95 | Barber 2016 | Include |  | November 2020 (MT checked by AWS/JN) |
| 96 | Freeman 2017 | Include |  | November 2020 (MT checked by AWS/JN) |
| 97 | Fucito 2017 | Include |  | November 2020 (MT checked by AWS/JN) |
| 98 | Gao 2014 | Include |  | November 2020 (MT checked by AWS) |
| 99 | Gellis 2013 | Include |  | November 2020 (MT checked by AWS/JN) |
| 100 | Gipson 2019 | Include |  | November 2020 (MT checked by AWS/JN) |
| 101 | Greeson 2014 | Include |  | November 2020 (MT checked by AWS/JN) |
| 102 | Hall 2018 | Include |  | November 2020 (MT checked by AWS/JN) |
| 103 | Hershner 2018 | Include |  | November 2020 (MT checked by AWS) |
| 104 | Jones 2019 | Include |  | November 2020 (MT checked by AWS) |
| 105 | Kloss 2015 | Include |  | November 2020 (MT checked by AWS) |
| 106 | Mairs 2015 | Include |  | November 2020 (MT checked by AWS) |
| 107 | Means 2000 | Include |  | November 2020 (MT checked by AWS) |
| 108 | Morris 2016 | Include |  | November 2020 (MT checked by AWS/JN) |
| 109 | Zhu 2024 | Include |  | September 2024 (MT checked by AWS/JN) |
| 110 | Classen 2022 | Include |  | November 2023 (MT checked by AWS/JN) |
|  |  |  |  |  |
